# Design and performance of the APPLE-Knot undulator

**DOI:** 10.1107/S1600577515006062

**Published:** 2015-06-09

**Authors:** Fuhao Ji, Rui Chang, Qiaogen Zhou, Wei Zhang, Mao Ye, Shigemi Sasaki, Shan Qiao

**Affiliations:** a State Key Laboratory of Functional Materials for Informatics, Shanghai Institute of Microsystem and Information Technology, Chinese Academy of Sciences, 865 Changning Road, Shanghai 200050, People’s Republic of China; b Department of Physics, State Key Laboratory of Surface Physics, and Laboratory of Advanced Materials, Fudan University, 2005 Songhu Road, Shanghai 200438, People’s Republic of China; c Shanghai Synchrotron Radiation Facility, Shanghai Institute of Applied Physics, Chinese Academy of Sciences, 239 Zhangheng Road, Shanghai 201204, People’s Republic of China; d Hiroshima Synchrotron Radiation Center, Hiroshima University, 2-313 Kagamiyama, Hiroshima 739-0046, Japan; f School of Physical Science and Technology, ShanghaiTech University, 319 Yueyang Road, Shanghai 200031, People’s Republic of China

**Keywords:** undulator, Knot undulator, figure-8 undulator, APPLE undulator, APPLE-8 undulator, Apple-Knot undulator

## Abstract

Along with the development of accelerator technology, synchrotron emittance has continuously decreased. This results in increased brightness, but also causes a heavy heat load on beamline optics. Recently, optical surfaces with 0.1 nm micro-roughness and 0.05 µrad slope error (r.m.s.) have become commercially available and surface distortions due to heat load have become a key factor in determining beamline performance, and heat load has become a serious problem at modern synchrotron radiation facilities. Here, APPLE-Knot undulators which can generate photons with arbitrary polarization, with low on-axis heat load, are reported.

## Abstract 2

The design and performance of the Apple-Knot undulator which can generate photons with arbitrary polarization and low on-axis heat load are presented.

## 1. Introduction   

A high-resolution photoelectron spectroscopy (HRPES) beamline has been constructed at the Shanghai Synchrotron Radiation Facility (SSRF). To achieve high resolution, the energy range of this beamline is hoped to be extended as low as 7 eV, because of the availability of laser sources below this energy. In this case, heat load becomes a challenging problem because an undulator with a deflection parameter *K* larger than 10 is required to generate 7 eV photons at SSRF, a 3.5 GeV storage ring. Due to relativistic effects, the radiation power from the synchrotron is emitted along the velocity direction of the electron beam (Hofmann, 2004 ▸), so that the heat-load problem is only an issue for linear undulators. For elliptical undulators, the electron velocity and heat load always deviate from the undulator axis, and, because the fundamental synchrotron radiation is emitted along the axis, most of the heat load can be blocked by an aperture on the axis.

Some novel undulators have been proposed to generate linearly polarized photons with low on-axis heat load. In a figure-8 undulator (Tanaka & Kitamura, 1995 ▸), the magnetic field period in the horizontal direction is twice as large as that in the vertical direction. The electrons move in a figure-eight orbit with alternating right- and left-handed circles, resulting in the cancellation of circular polarization and a remnant linear polarization. The disadvantage of figure-eight undulators is their incapability to generate circularly polarized light. A Knot undulator (Qiao *et al.*, 2009 ▸; Xi *et al.*, 2013 ▸), based on electromagnets, has been proposed that can generate photons with arbitrary polarization with low on-axis heat load. The electromagnetic undulator offers the theoretical possibility of fast polarization switching of photons without any mechanical motion, which is important for dichroism measurements and has been used successfully at the DESIRS beamline at SOLEIL (Nahon *et al.*, 2012 ▸). However, the hysteresis of the electromagnets is harmful to the stable operation of the storage ring, and electromagnets are power-wasting. Hence, an undulator with permanent magnets that can maintain the advantages of the Knot type is highly desired.

## 2. Apple-Knot undulator inheriting from Apple-8 structure   

**Figure 1:**
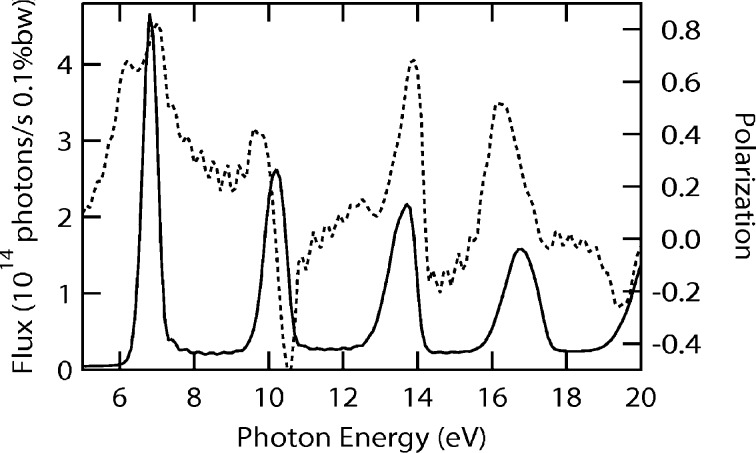
The intensity (left axis, solid line) and linear polarization (right axis, broken line) of photons from a typical figure-8 undulator of Shanghai Synchrotron Radiation Facility, a 3.5 GeV ring of 300 mA beam current, with 0.6 mrad collection angles in both vertical and horizontal directions.

An APPLE-8 undulator (Sasaki *et al.*, 1998 ▸) which can generate photons with arbitrary polarization, a hybrid of both APPLE (Sasaki *et al.*, 1993 ▸; Sasaki, 1994 ▸) and figure-8 undulators, can seemingly overcome all the problems mentioned above. However, a figure-8 undulator has a fundamental defect in that it cannot generate photons with pure linear polarization because of the two-to-one period ratio: the second-harmonic energy of the vertically polarized photons generated by the horizontal magnetic field is always the same as that of the fundamental photons with horizontal polarization. Although the on-axis photons have pure horizontal polarization because of the lack of even harmonics along the axis, the practical linear polarization degrades as the collection angle increases. From the consideration of spatial coherence (diffraction limit), the r.m.s. of size σ and divergence angle σ′ of the fundamental photons from the undulator are (Onuki & Elleaume, 2003 ▸)
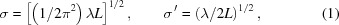
where λ and *L* are the photon wavelength and total length of the undulator, respectively. For a 4.4 m undulator, the beam divergence of 7 eV photons, that is 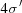 in which 95% photons are included, is about 0.6 mrad. The intensity and linear polarization of photons from a typical 22-period 4.4 m figure-8 undulator are shown in Fig. 1 ▸. In this paper, all the calculations of photon flux, photon polarization and heat load from undulators were carried out using the *SPECTRA* program (Tanaka & Kitamura, 2001 ▸). In the calculations, the amplitudes of the vertical and horizontal magnetic fields are 0.5 T and 0.235 T, respectively. The linear polarization (defined as 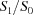 from Stoke parameters 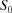 and 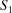) at the peak intensity around 6.8 eV is only 74.9%. The distinguishing feature of the Knot-type undulator is the two to three period ratio of the vertical and horizontal magnetic fields, and thus it can avoid the inherent low polarization defect of the figure-8 undulator.

**Figure 2:**
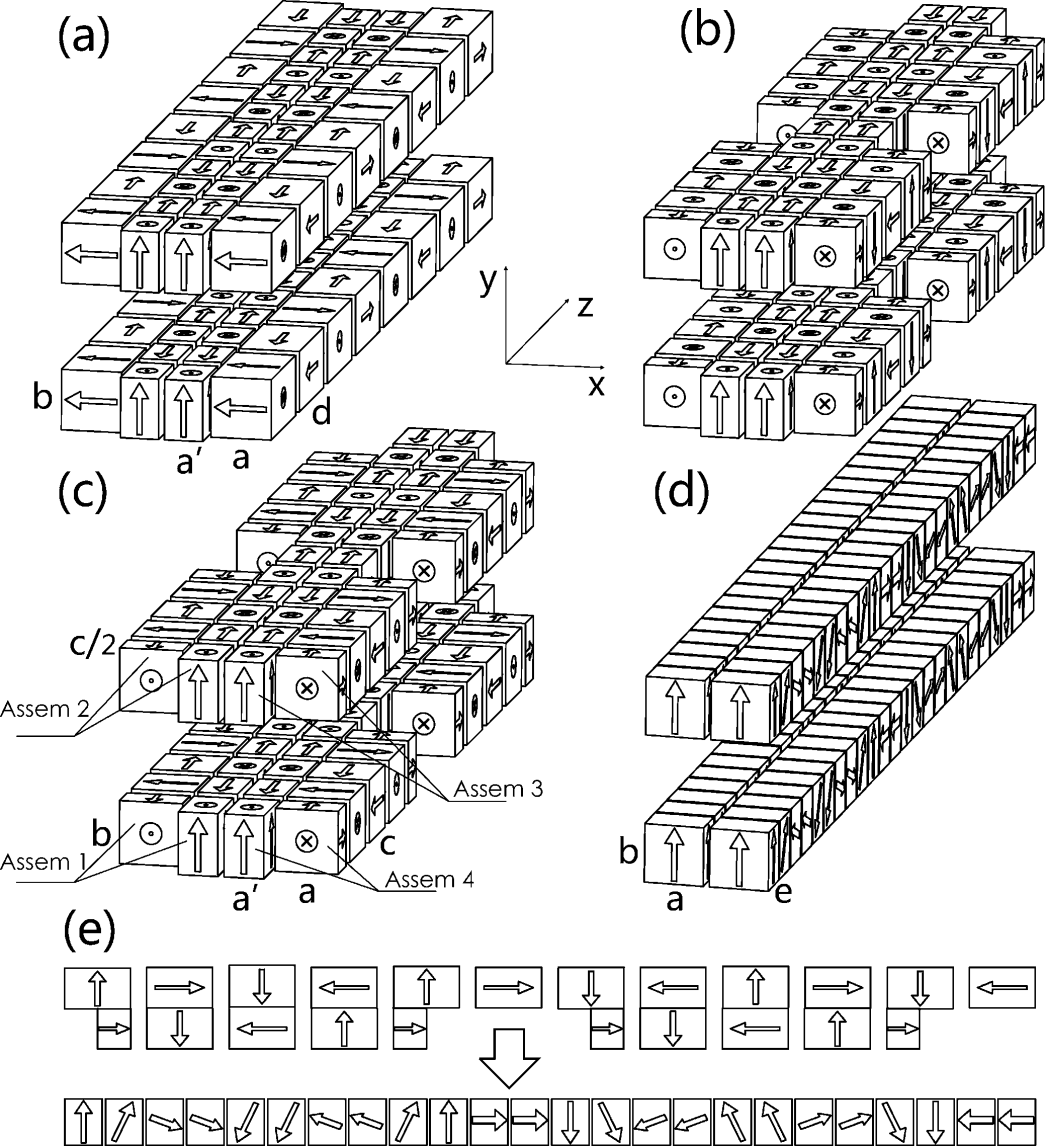
Different APPLE-Knot structures. (*a*) Inheriting from APPLE-8 undulator. (*b*) Proposed by Sasaki *et al.* (2013 ▸). (*c*) Proposed by this work. (*d*) Combined magnets. (*e*) Combination process.

**Figure 3:**
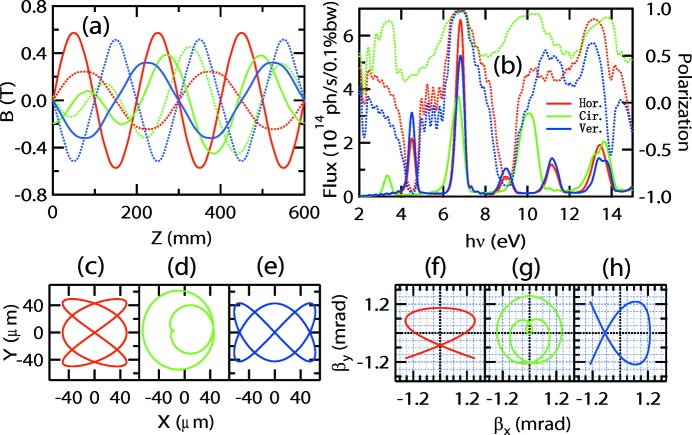
Performance of the APPLE-Knot undulator with Fig. 2(*a*) ▸ structure in horizontal (red), circular (green) and vertical (blue) modes. (*a*) Magnetic fields along the *x* (solid lines) and *y* (broken lines) directions. (*b*) Intensities (left axes) and polarizations (right axes) of photons at different energies. (*c*), (*d*), (*e*) Electron orbits and (*f*), (*g*), (*h*) electron velocities in the horizontal, circular and vertical modes, respectively.

A simple and direct method to construct an APPLE-Knot undulator is to change the one to two period ratio of the APPLE-8 to two to three by combining two standard APPLE undulators (Fig. 2*a* ▸). The inner APPLE undulator with four magnet rows is responsible for the generation of photons with arbitrary polarization and the outer four rows are used to deflect the electron beam to move in a knot orbit. The geometry parameters of magnets in Fig. 2(*a*) ▸ are *a* = *b* = 35 mm, *a*′ = 22 mm and *d* = 65 mm. The clearances along the *x* direction are 3.5 mm between adjacent APPLE rows and 2 mm between adjacent APPLE and Knot rows. The clearance along the *z* direction is 10 mm between all adjacent magnets. The related magnetic fields in different modes are shown in Fig. 3(*a*) ▸. All the magnetic fields shown in this paper were calculated using the *RADIA* magnetostatics program (Chubar *et al.*, 1998 ▸). To generate horizontal (vertical) polarized photons, the phases between different rows are adjusted so that the inner and outer APPLE rows generate magnetic fields along the vertical (horizontal) and horizontal (vertical) directions only. To generate circularly polarized photons, the phases are adjusted so that both the inner and outer APPLE undulators are in circular mode with different periods. The performance is shown in Fig. 3 ▸. The horizontal, vertical and circular polarizations are defined as 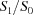, −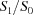 and 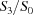 from Stoke parameters 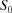, 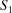 and 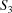. For both horizontal (Fig. 3*f* ▸) and vertical (Fig. 3*h* ▸) modes, the velocities of electrons deviate from the undulator axis with a larger than 0.3 mrad angle and only small heat load remains inside the 0.6 mrad acceptance angle. However, part of the velocity locates in the 0.6 mrad acceptance angle for circularly polarized mode. Satisfactory performance can be achieved with heat loads of 31, 55 and 37 W for horizontal, circular and vertical modes inside the 0.6 mrad × 0.6 mrad acceptance solid angle, respectively, compared with 1106 W for a pure linear undulator. The only problem with this type of APPLE-Knot structure is the requirement for relative movement between all the magnet rows, which is not easy to implement.

## 3. Apple-Knot undulator with blank segments   

**Figure 4:**
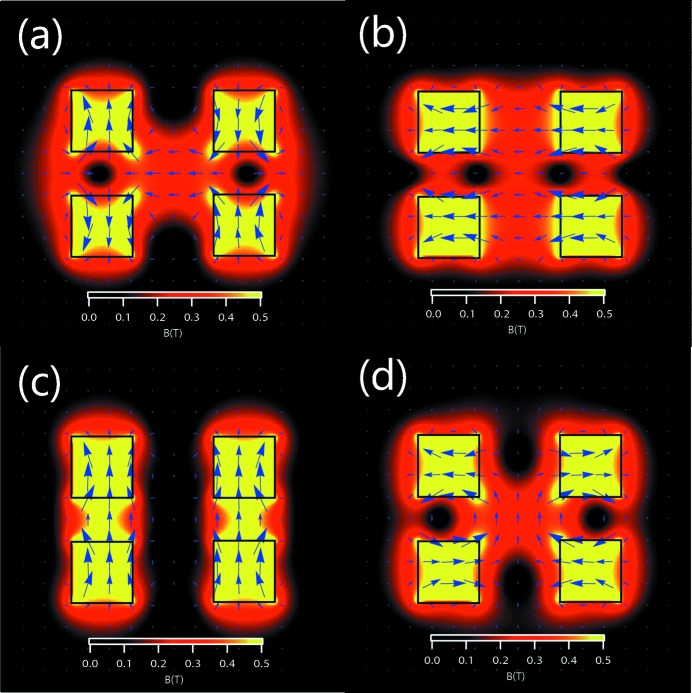
(*a*), (*b*) [(*c*), (*d*)] Horizontal [vertical] magnetic intensities generated by the Knot rows in the undulators with Fig. 2(*b*) and Fig. 2 ▸(*c*) structures in horizontal [vertical] mode.

Sasaki *et al.* (2013 ▸) suggested an APPLE-Knot undulator (Fig. 2*b* ▸) combining a standard inner APPLE configuration and an outer Knot structure with blank segments, imitating the electromagnetic Knot undulator (Qiao *et al.*, 2009 ▸). The undulator consists of four assemblies. Each of them is composed of one APPLE row and one Knot row with a fixed relative position. Switching between different operating modes is by relative movement of the different assemblies. The geometry parameters of magnets in Figs. 2(*b*) and 2(*c*) ▸ are *a* = *b* = 35 mm, *a*′ = 22 mm and *c* = 40 mm. The clearances along the *x* direction are 3.5 mm between adjacent APPLE rows and 2 mm between adjacent APPLE and Knot rows. The clearance along the *z* direction is 10 mm between all adjacent magnets. The problem with Sasaki’s design is the too weak magnetic field generated by Knot rows in vertical mode, resulting in the ineffective suppression of on-axis heat load. Although in horizontal mode its Knot rows can generate a horizontal magnetic field around the axis (Fig. 4*a* ▸), because of the large horizontal gaps, in vertical mode the magnetic field along the vertical direction will concentrate around the magnetic poles, resulting in low magnetic intensity around the undulator axis (Fig. 4*c* ▸).

**Figure 5:**
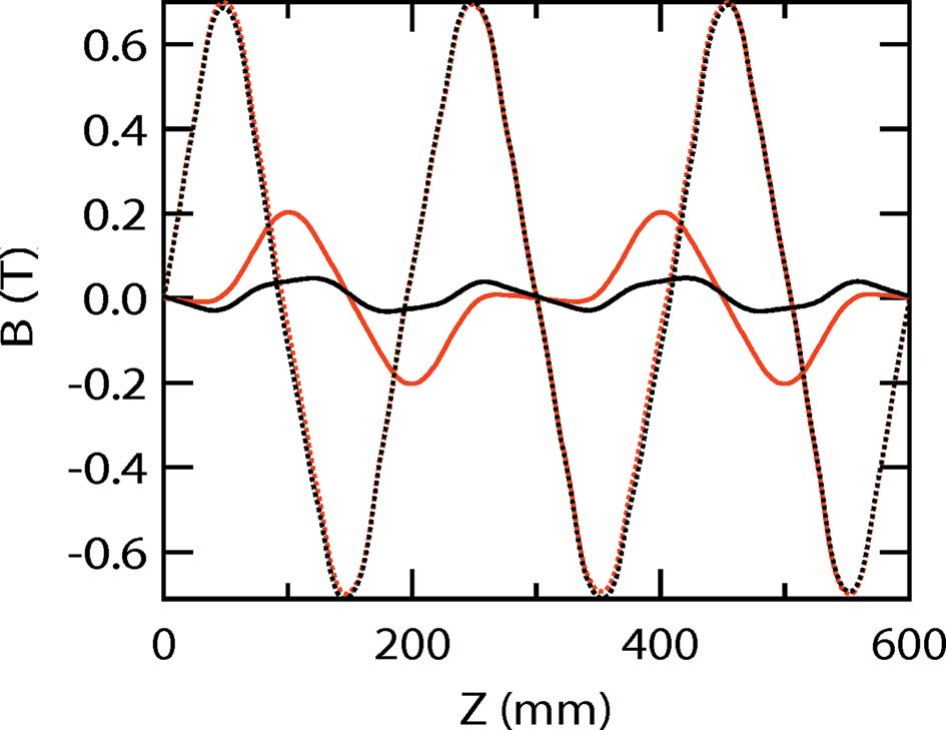
Comparison of the vertical (solid lines) and horizontal (broken lines) magnetic fields of APPLE-Knot undulators with Figs. 2(*b*) (black) and 2(*c*) ▸ (red) structures in vertical mode.

One method to overcome this problem is to rotate the magnetization direction of the vertical magnetized magnets in the Knot rows by 90° to horizontal (Fig. 2*c* ▸). Then, the horizontal magnetic intensity around the axis generated by the Knot rows in horizontal mode (Fig. 4*b* ▸) is strong enough because of the small vertical gap, and that along the vertical direction in vertical mode (Fig. 4*d* ▸) can recover to a certain extent because of the opposite magnetization of adjacent Knot magnets, which forces the magnetic field to go across the axis. A comparison of the magnetic fields of the structures in Figs. 2(*b*) and 2(*c*) ▸ in vertical mode is shown in Fig. 5 ▸. The magnetic field in the vertical direction for the structure in Fig. 2(*c*) ▸ is clearly enhanced, whereas the magnetic fields in the horizontal direction are almost the same.

**Figure 6:**
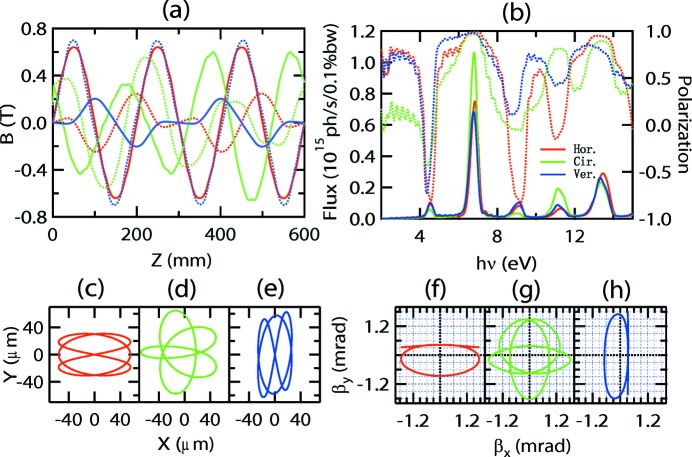
Performance of the undulator with structure shown in Fig. 2(*c*) ▸ in horizontal (red), circular (green) and vertical with antiparallel shifts (blue) modes. (*a*) Vertical (solid line) and horizontal (broken line) magnetic fields. (*b*) Fluxes (left axis, solid line) and linear or circular polarizations (right axis, broken line) at different photon energies. (*c*), (*d*), (*e*) Electron orbits and (*f*), (*g*), (*h*) electron velocities in horizontal, circular and vertical modes, respectively.

With the configuration shown in Fig. 2(*c*) ▸, the APPLE (Knot) rows generate a magnetic field along the vertical (horizontal) direction only, and the undulator works in the horizontal mode to generate fundamental photons with horizontal polarization. The magnetic fields, electron beam orbit, electron beam velocity and related photon flux and polarization are shown in Figs. 6(*a*), 6(*c*), 6(*f*) and 6(*b*) ▸, respectively. A 99.3% horizontal linear polarization is achieved with peak intensity at 6.85 eV. The electron beam velocity always deviated from the undulator axis by more than 0.3 mrad and the heat load inside the 0.6 mrad × 0.6 mrad acceptance solid angle is 59.4 W.

To generate circularly polarized photons, assemblies 1 and 3 [see Fig. 2(*c*) ▸] need to shift by 47.5 mm relative to assemblies 2 and 4. The magnetic fields, electron beam orbit, electron beam velocity, photon flux and circular polarization are shown in Figs. 6(*a*), 6(*d*), 6(*g*) and 6(*b*) ▸, respectively. A 97% circular polarization is achieved with the peak flux at 6.81 eV. The velocity always deviates from the undulator axis by more than 0.3 mrad and the heat load is 43.9 W inside the 0.6 mrad × 0.6 mrad acceptance solid angle.

**Figure 7:**
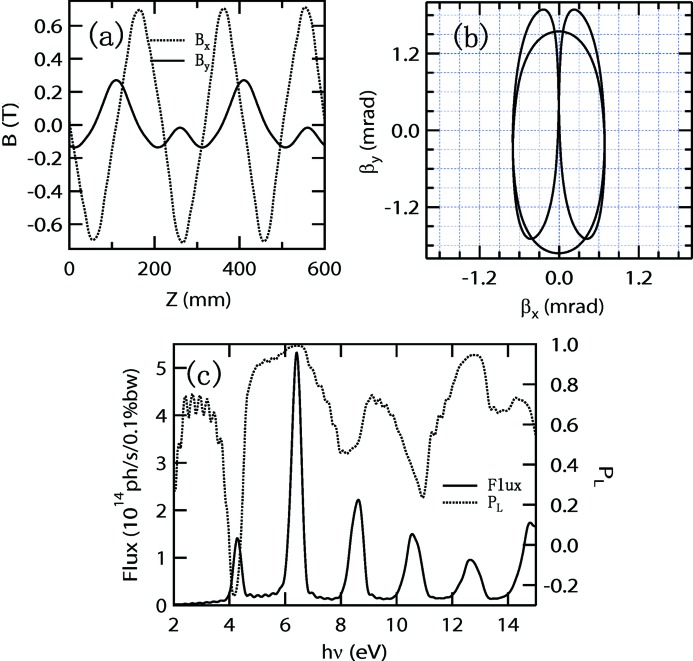
Magnetic field (*a*), electron velocity (*b*), photon flux and vertical polarization (*c*) of the undulator with Fig. 2(*c*) ▸ structure by parallel π phase shift.

There are two methods of generating vertically polarized photons. The first method is to shift assemblies 1 and 3 by λ_u_/2 (100 mm, π phase, where λ_u_ is the period of the APPLE rows) relative to assemblies 2 and 4. The magnetic fields, electron beam velocity, photon flux and vertical linear polarization are shown in Fig. 7 ▸. A 99% vertical polarization is achieved with peak flux at 6.4 eV. From the velocity map, the velocity is not always deviated from the undulator axis, resulting in a heat load of 314 W inside the 0.6 mrad × 0.6 mrad acceptance solid angle.

To obtain a smaller on-axis heat load, Sasaki suggested shifting assemblies 1 and 3 along the *z* axis by λ_u_/2 and −λ_u_/2, respectively (Sasaki *et al.*, 2013 ▸). In this case, although the magnetic field generated by the APPLE rows is the same as that with a parallel movement, the field from the Knot rows changes. The magnetic fields, electron beam orbit, electron beam velocity, photon flux and vertical linear polarization by these antiparallel shifts are shown in Figs. 6(*a*), 6(*e*), 6(*h*) and 6(*b*) ▸, respectively. A 97.6% vertical polarization is achieved with peak flux at 6.77 eV. The velocity is always deviated from the undulator axis by more than 0.3 mrad and the heat load is 178 W inside the 0.6 mrad × 0.6 mrad acceptance solid angle.

It is interesting that the phase differences between magnetic fields along the horizontal and vertical directions are π/2 or zero, respectively, for parallel or antiparallel shifts. Therefore, it is possible to generate linearly polarized photons with arbitrary angles between the polarization and the horizon by antiparallel shifts. To explain the effect clearly, the magnetic fields generated by different configurations of APPLE rows need to be estimated. The vector potential of magnetic moment 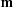 is
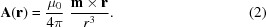
The magnetic field 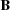 generated by 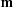 is
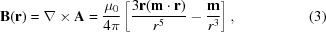
which can be divided into two parts, one along the 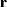 direction and another along the 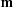 direction, so the on-axis magnetic fields generated by APPLE rows in assembly 2 and 4 are
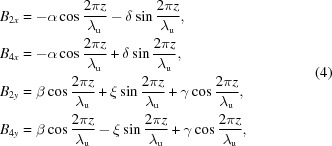
where the α, β, δ and ξ terms are related to the 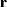 part and the γ term is related to the 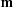 part. Similarly, the magnetic fields generated by APPLE rows in assembly 1 and 3 are
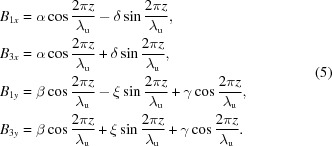
The magnetic fields generated by APPLE rows in assembly 1 and 3 after they shift the same *D* along *z* can be estimated as
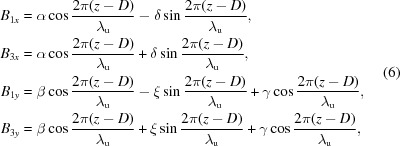
and the total magnetic fields are
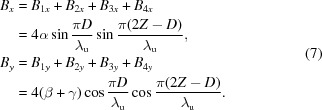
In this case, the magnetic fields 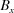 and 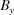 always differ by 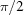 in phase and the photons generated by the APPLE rows are usually elliptically polarized. The polarization becomes horizontally or vertically linear when *D* = 0 or λ_u_/2, respectively.

The magnetic fields generated by APPLE rows in assembly 1 and 3 after they shift *D* and −*D* along *z* can be estimated as
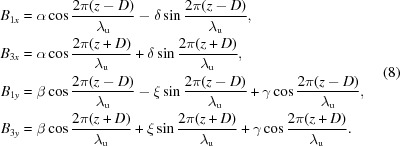
The total magnetic fields are
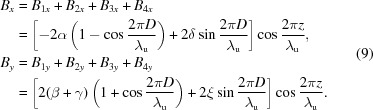
Because of the zero phase difference between 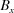 and 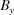, the photons generated with APPLE rows with antiparallel movement are linearly polarized and the polarization angle can be adjusted through the adjustment of the *D* value.

**Figure 8:**
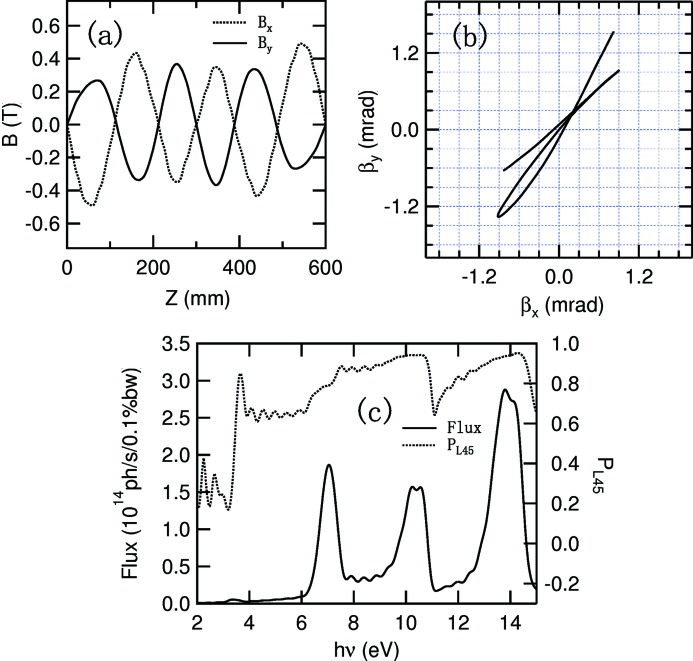
Magnetic field (*a*), electron velocity (*b*), photon flux and 45° polarization (*c*) of the undulator with Fig. 2(*c*) ▸ structure in 45° linear polarization mode.

Linearly polarized photons 45° to the horizon can be generated after a 55 mm antiparallel movement of assemblies 1 and 3 in the undulator with the Fig. 2(*c*) ▸ structure. The magnetic fields, electron beam orbit and related photon fluxes and 45° linear polarization (defined as 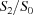 from Stoke parameters 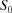 and 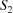) are shown in Fig. 8 ▸. The 45° polarization is 94.2% with peak flux of fundamental photons at 10.3 eV. From the electron beam velocity map we can see that the electron beam has an almost linear motion, resulting in the loss of heat load suppression function. However, the antiparallel movement provides the possibility to generate linearly polarized photons with arbitrary angle, important for experiments to study the symmetry of matter. An APPLE-Knot undulator with structure shown in Fig. 2(*c*) ▸ is being constructed at SSRF and performance tests will be carried out before the middle of 2016.

## 4. Apple-Knot undulator with merged magnets   

**Figure 9:**
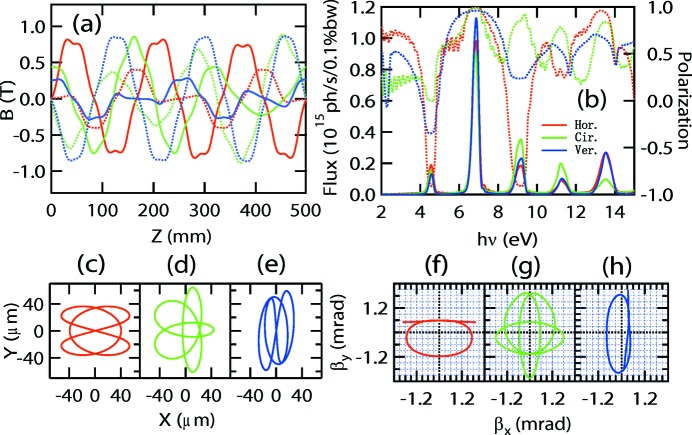
Performance of combined magnets shown in Fig. 2(*d*) ▸ in horizontal (red), circular (green) and vertical (blue) modes. (*a*) Vertical (solid line) and horizontal (broken line) magnetic fields. (*b*) Fluxes (left axis, solid line) and linear or circular polarizations (right axis, broken line) at different photon energies. (*c*), (*d*), (*e*) Electron orbits and (*f*), (*g*), (*h*) electron velocities in horizontal, circular and vertical modes, respectively.

Another way to resolve the problem of the weak Knot fields is to superimpose the APPLE and Knot rows as shown in Fig. 2(*e*) ▸; that is, by vector addition of the magnetization and normalization of the final amplitude to the saturated value of the magnet material. Then the APPLE-Knot undulator recovers the standard APPLE four-row structure (Fig. 2*d* ▸) and the change is only the rotations of magnetic orientation. The ratio of the main (APPLE) and auxiliary (Knot) magnetic fields can be adjusted by the rotation angles, enabling the adjustment of the electron beam orbit to generate bright photons with acceptable heat load. A simple policy is that the electron beam should be deflected by more than half the divergence of the photons; then most of the heat load can be blocked by the white-light aperture in the beamline. The geometry parameters of magnets in Fig. 2(*d*) ▸ are *a* = *b* = 35 mm and *e* = 18.75 mm. The clearances are 3.5 mm and 2 mm between adjacent magnets along the *x* and *z* directions. The ratio of magnetization of APPLE and Knot magnets is chosen as 1.96 which corresponds to a typical rotation angle of 27°. The magnetic fields, electron beam orbits, photon fluxes and polarizations of the APPLE-Knot undulator with combined magnets in different modes are shown in Fig. 9 ▸. The horizontal, circular and vertical polarizations are 99%, 99% and 96% with corresponding heat loads of 13.2 W, 25.4 W and 233 W, respectively. Because of the stronger magnetic field, the period of combined magnets is shorter and more periods can be included in the straight section, resulting in higher flux and corresponding higher heat load compared with the structure of Fig. 2(*c*) ▸.

**Figure 10:**
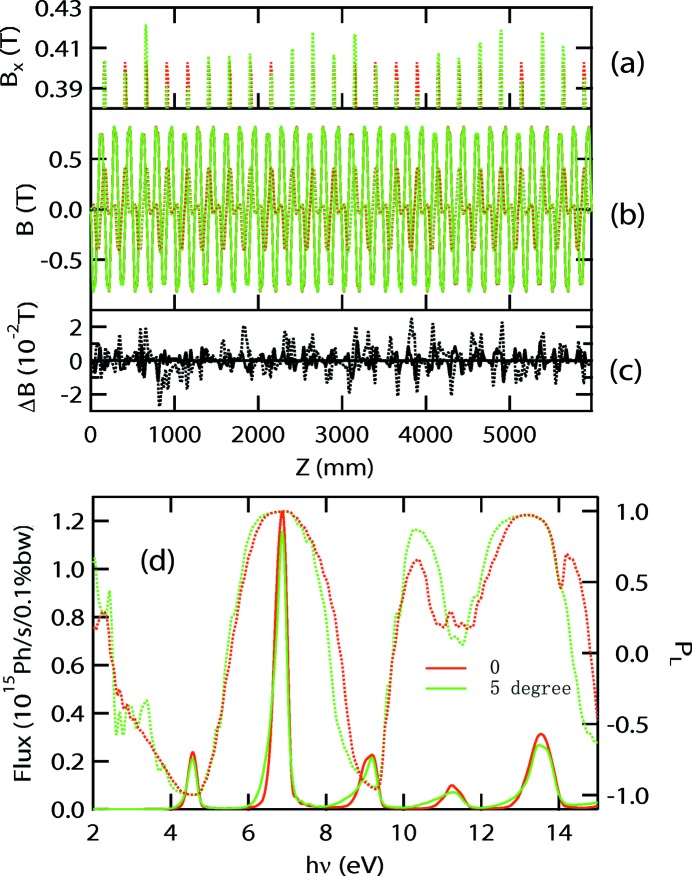
Performance of the APPLE-Knot undulator with Fig. 2(*d*) ▸ structure with 0° (red) and 5° (green) orientation errors. (*a*) Zoom of the horizontal magnetic fields. (*b*) Horizontal (broken lines) and vertical (solid lines) magnetic fields. (*c*) Difference of vertical (solid line) and horizontal (broken line) magnetic fields of 0° and 5° orientation errors. (*d*) Photon fluxes (solid line) and linear polarizations (broken lines).

One concern about this structure is the practical accuracy of the magnetic orientation of the magnets. Magnets with 1° orientation accuracy are commercially available. To estimate the effect of the orientation error, the performance of the APPLE-Knot undulator with Fig. 2(*d*) ▸ structure in horizontal mode with 5° orientation error was simulated. In the *RADIA* program, the magnetization direction of the magnet is characterized by a vector (*a*, *b*, *c*) and the typical orientation vector of a magnet inside the Fig. 2(*d*) ▸ structure can be represented by (0, cos27, sin27). To determine the performance of the undulator with a random 5° orientation error, two uniform distributed random numbers *r*1 and *r*2 in the (−2.5, 2.5) range were generated for each magnet and the orientation vectors of every magnet were adjusted according to the random numbers. For example, (0, cos27, sin27) was adjusted to [sin(*r*1), cos(*r*1)cos(27 + *r*2), cos(*r*1)sin(27 + *r*2)]. After designating the initial magnetic orientations of all magnets, a relaxation process was carried out to consider the magnetic interactions between segments. The magnetic fields, photon fluxes and polarizations with 0 and 5° orientation errors are shown in Fig. 10 ▸. The polarizations are almost unchanged and the flux only decreases by 7% with a 5° orientation error.

## 5. Conclusion   

In conclusion, the proposed novel APPLE-Knot undulator structures can supply photons with arbitrary desired polarization and thoroughly resolve the heat-load problem, a serious issue for modern synchrotron radiation facilities. Our discovery extends the low-energy limitation of synchrotron radiation sources and opens the possibility to construct beamlines with good performance which were unavailable before.
